# Bradykinin‐mediated Ca^2+^ signalling regulates cell growth and mobility in human cardiac c‐Kit^+^ progenitor cells

**DOI:** 10.1111/jcmm.13706

**Published:** 2018-08-17

**Authors:** Gang Li, Hui Che, Wei‐Yin Wu, Ling‐Jun Jie, Guo‐Sheng Xiao, Yan Wang, Gui‐Rong Li

**Affiliations:** ^1^ Xiamen Cardiovascular Hospital Xiamen University Xiamen Fujian China; ^2^ Department of Medicine Li Ka Shing Faculty of Medicine University of Hong Kong Pokfulam Hong Kong China

**Keywords:** bradykinin, cell cycle progression, human cardiac c‐Kit^+^ progenitor cells, inositol 1,4,5‐triphosphate receptor, migration, store‐operated Ca^2+^ entry

## Abstract

Our recent study showed that bradykinin increases cell cycling progression and migration of human cardiac c‐Kit^+^ progenitor cells by activating pAkt and pERK1/2 signals. This study investigated whether bradykinin‐mediated Ca^2+^ signalling participates in regulating cellular functions in cultured human cardiac c‐Kit^+^ progenitor cells using laser scanning confocal microscopy and biochemical approaches. It was found that bradykinin increased cytosolic free Ca^2+^ (${\mathrm{Ca}}_{i}^{2+}$) by triggering a transient Ca^2+^ release from ER IP3Rs followed by sustained Ca^2+^ influx through store‐operated Ca^2+^ entry (SOCE) channel. Blockade of B2 receptor with HOE140 or IP3Rs with araguspongin B or silencing IP3R3 with siRNA abolished both Ca^2+^ release and Ca^2+^ influx. It is interesting to note that the bradykinin‐induced cell cycle progression and migration were not observed in cells with siRNA‐silenced IP3R3 or the SOCE component TRPC1, Orai1 or STIM1. Also the bradykinin‐induced increase in pAkt and pERK1/2 as well as cyclin D1 was reduced in these cells. These results demonstrate for the first time that bradykinin‐mediated increase in free ${\mathrm{Ca}}_{i}^{2+}$ via ER‐IP3R3 Ca^2+^ release followed by Ca^2+^ influx through SOCE channel plays a crucial role in regulating cell growth and migration via activating pAkt, pERK1/2 and cyclin D1 in human cardiac c‐Kit^+^ progenitor cells.

## 1 INTRODUCTION

Cardiac progenitor cells, also known as cardiac stem cells, are self‐renewing, clonogenic and pluripotent and can differentiate into a minimum of three cell types including cardiomyocytes, vascular smooth muscle cells and vascular endothelial cells.^1^ Among cardiac progenitor cells, c‐Kit^+^ progenitor cells are a widely studied cell type for myocardial repair. They reconstitute the myocardium with new vessels and myocytes after being injected into the ischaemic heart.^2^, ^3^ Therefore, the cardiac c‐Kit^+^ progenitor cell is believed to be a cell source for transplantation to treat ischaemic cardiomyopathy. A clinical trial showed that the cardiac c‐Kit^+^ progenitor cells isolated from patients with ischaemic cardiomyopathy may significantly improve heart function and the quality of life when transplanted back into the patients via intracoronary injection;^4^, ^5^ however, current cell therapy with adult cardiac c‐Kit^+^ progenitor cells for ischaemic cardiomyopathy is limited by the poor survival and retention in the heart and the lack of sufficient de novo differentiation of transplanted stem cells into mature cardiac cell types,^6^, ^7^, ^8^ which is at least in part due to the poor understanding of cellular biology and physiology of cardiac c‐Kit^+^ progenitor cells.

An earlier study reported that spontaneous oscillations of cytosolic free Ca^2+^ (${\mathrm{Ca}}_{i}^{2+}$) play an important role in regulating human cardiac c‐Kit^+^ progenitor cell growth.^9^ Our recent study demonstrated that the endogenous peptide bradykinin promotes cell cycling progression and migration via activating PI3K, phospholipase C (PLC), pAkt, pERK1/2 and cyclin D1 in human cardiac c‐Kit^+^ progenitor cells.^10^ However, it is unknown whether bradykinin‐induced cycle progression and migration are related to Ca^2+^ signalling in human cardiac c‐Kit^+^ progenitor cells. This study was therefore designed to investigate the detailed molecular mechanisms of whether/how Ca^2+^ signalling‐related channels/receptors are involved in bradykinin‐induced increase in cell growth and migration in human cardiac c‐Kit^+^ progenitor cells with multiple biochemical/molecular biological approaches. Our results demonstrated that bradykinin‐induced ${\mathrm{Ca}}_{i}^{2+}$ increase by activating Ca^2+^ release via inositol triphosphate receptor 3 (IP3R3) followed by Ca^2+^ influx through store‐operated Ca^2+^ entry (SOCE) channel, which plays a crucial role in regulating cell cycle progression and migration via activating pAkt, pERK1/2 and cyclin D1 in human cardiac c‐Kit^+^ progenitor cells.

## 2 MATERIALS AND METHODS

### 2.1 Experimental solutions and reagents

The experimental solutions and reagents used in this study were described in online Supporting Information (Supplementary Materials and Methods).

### 2.2 Cell culture

Human cardiac c‐Kit^+^ progenitor cells were isolated from human atrial specimens from patients undergoing coronary artery bypass surgery as described previously.^11^, ^12^, ^13^, ^14^ The tissue collection was approved by the Ethics Committee of the University of Hong Kong (UW‐10‐174) with patients’ consent. The study conforms with the declaration of Helsinki the Declaration of Helsinki (see Cardiovascular Research 1997;35:2‐4) for using human tissue. The cells were maintained in α‐MEM supplemented with 15% FBS, 2 mmol L^−1^ l‐glutamine, 5 ng/mL bFGF, 5 ng/mL EGF, 100 U/mL penicillin and 100 μg/mL streptomycin in a humidified atmosphere of 5% CO_2_ at 37°C. The cells at 3‐6 passages used in this study were from 2 female patients (54 and 56 years old) and 2 male patients (48 and 61 years old).

### 2.3 Cytosolic Ca^2+^ measurement

Cytosolic free Ca^2+^ (${\mathrm{Ca}}_{i}^{2+}$) was measured with a laser scanning confocal microscopy technique in human cardiac c‐Kit^+^ progenitor cells loaded with the free Ca^2+^ indicator Fluo‐4 AM (Thermo Fisher Scientific Inc.) following manufacturer's instruction.^13^ Briefly, cells were seeded on 35‐mm plates at a density of approximately 1 × 10^4^ cells for 24 hours, and then loaded with 1 μmol L^−1^ Fluo‐4 AM in FBS‐free α‐MEM for 30 minutes at 37°C in the dark. Afterwards, the cells were washed three times with Tyrode's solution to remove the residual fluorescent dye. ${\mathrm{Ca}}_{i}^{2+}$ was monitored every 5 seconds using the laser scanning confocal microscope Leica SP5‐II at room temperature (23‐25°C).

### 2.4 Small interfering RNA

Gene silencing was conducted with small interfering RNA (siRNA) technique as described previously.^11^, ^13^ Briefly, human cardiac c‐Kit^+^ progenitor cells were seeded in six‐well plates or 96‐well plates at a confluence of 60%‐80% overnight. Then the cells were transfected with different siRNA molecules (Santa Cruz Biotech) at 10 or 40 nmol L^−1^ using Lipofectamine 2000 reagent (Thermo Fisher Scientific) for 48‐72 hours. The control siRNA, which had no known target in the human genome, was used as negative control.

### 2.5 Reverse transcription‐polymerase chain reaction

Reverse transcription‐polymerase chain reaction was employed to determine mRNA expression in cells with silenced IP3Rs, TRPC channels or SOCE channels for siRNA efficacy as described previously.^10^, ^13^ Briefly, total RNA was extracted from human cardiac c‐Kit^+^ progenitor cells transfected with corresponding siRNA for 48 hours using TRIzol reagent. The amount of total RNA was quantified by spectrophotometry, and reverse transcription reaction was performed using 2 μg of total RNA to transcribe into complementary DNA with Advantage^®^ RT‐for‐PCR Kit (Takara biotech Co., Ltd, Dalian, China) following manufacturer's instruction. Primers for the corresponding targets are shown online in Supporting Information (Table S1).

### 2.6 Cell proliferation assay

Cell proliferation was detected with 3‐(4,5‐dimethyl‐2‐thiazolyl)‐2,5‐diphenyltetrazolium bromide (MTT) and 5‐bromo‐2‐deoxy uridine (BrdU) in human cardiac c‐Kit^+^ progenitor cells transfected with siRNAs targeting IP3Rs, TRPCs and SOCEs for 60 hours as described previously^11^, ^12^, ^13^, ^14^ and online in Supporting Information (Materials and Methods).

### 2.7 Flow cytometry analysis

The cell cycle distribution involved in the proliferation process was detected by flow cytometry in human cardiac c‐Kit^+^ progenitor cells as described previously.^11^, ^12^, ^13^, ^14^ Briefly, cells were dissociated with 0.25% trypsin, washed three times with phosphate‐buffered saline (PBS) and fixed with cold 70% ethanol at 4°C over night. The ethanol was removed by centrifuge, and the cell pellets were washed with PBS for three times. Then, the propidium iodide/PBS staining buffer (propidium iodide 20 μg/mL, RNase A 10 μg/mL and 0.1% Triton‐X 100) was used to stain the cells at 37° for 30 minutes. Data were acquired with a Beckman Coulter FC500, and the percentages of G0/G1‐phase, S‐phase and G2/M‐phase cells were calculated with MODFIT LT software (BD Biosciences, San Jose, CA, USA).

### 2.8 Cell mobility assay

The effects of bradykinin on human cardiac c‐Kit^+^ cells transfected with corresponding siRNA were determined with wound‐healing and transwell assay as described previously^11^, ^12^, ^13^, ^14^ and online in Supporting Information (Materials and Methods).

### 2.9 Western blot analysis

Western blot was conducted to determine the protein expression in human cardiac c‐Kit^+^ progenitor cells as described previously.^10^ The silencing efficiency of IP3Rs, TRPC channels, STIM1 and Orai1 was determined in cells transfected with the corresponding siRNAs for 72 hours, and cyclin D1, Akt and ERK1/2 as well as their phosphorylated levels were determined in these cells. Briefly, the cells were lysed with RIPA buffer and mixed with sample buffer, heated to 95°C for at least 5 minutes and cooled on ice. Samples were electrophoresed on SDS‐PAGE gels and then transferred onto PVDF membranes (Bio‐Rad, Hercules, CA, USA). The membranes were blocked with 5% non‐fat milk or 5% bovine serum albumin in Tween‐20 Tris‐buffer saline buffer (TTBS). Then, the membranes were incubated with corresponding primary antibodies at dilutions 1:1000 to 1:2000 in blocking buffer at 4°C overnight with agitation. After washed with TTBS, the membranes were then incubated with HRP‐conjugated secondary antibody at dilutions 1:5000‐1:10 000 at room temperature for 1 hour with agitation. The membranes were then washed 3 times with TTBS. Electrogenerated chemiluminescence was applied to develop X‐ray film with the membranes. The relative band intensities of Western blots were measured by quantitative scanning densitometry and the image analysis using Image J (https://imagej.nih.gov/ij/).

### 2.10 Statistical analysis

All data were presented as mean ± SEM. Unpaired Student's *t* test was used as appropriate to evaluate the statistical significance of differences between two group means. One‐way ANOVA followed by Tukey's test was applied for multiple group comparison. A value of *P* < .05 was considered to indicate statistical significance.

## 3 RESULTS

### 3.1 Effects of bradykinin on ${\mathrm{Ca}}_{i}^{2+}$ in human cardiac c‐Kit^+^ progenitor cells

In our previous study, bradykinin at 10 nmol L^−1^ remarkably stimulates cell growth and migration.^10^ We therefore utilize the 10 nmol L^−1^ bradykinin in this study to investigate whether the stimulation of cell growth and migration is linked to Ca^2+^ signalling induced by bradykinin.

**Figure 1:**
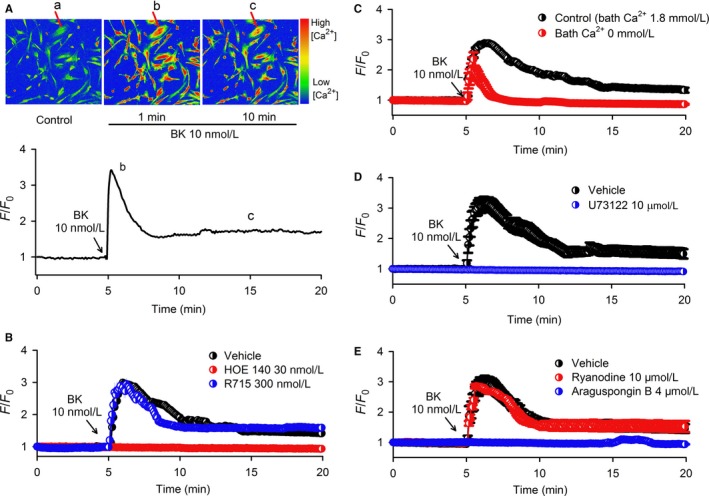
Increase in ${\mathrm{Ca}}_{i}^{2+}$ by bradykinin (BK) in human cardiac c‐kit^+^ progenitor cells. A, Pseudocolour images (upper panel) showing changes in fluorescence intensity (ie ${\mathrm{Ca}}_{i}^{2+}$) induced by 10 nmol L^−1^ bradykinin at different time points indicated in the lower panel in human cardiac c‐Kit^+^ progenitor cells. The F/F_0_ represents ${\mathrm{Ca}}_{i}^{2+}$ level, where the F is the changes in cell fluorescence intensity of Fluo‐4 AM, and the F_0_ is the initial level of fluorescence intensity. B, ${\mathrm{Ca}}_{i}^{2+}$ increase induced by bradykinin was prevented in cells pre‐treated with 30 nmol L^−1^ HOE140 (B2R inhibitor) (n = 80 cells of 5 experiments), but not the B1R inhibitor R715 (300 nmol L^−1^) (n = 80 cells of 5 experiments). C, Only a transient ${\mathrm{Ca}}_{i}^{2+}$ increase induced by bradykinin was observed in cells exposure to Ca^2+^‐free Tyrode's solution with 1 mmol L^−1^ EGTA (n = 96 cells of 6 experiments). D, Bradykinin‐induced Ca^2+^ increase was absent in cells pre‐treated with the PLC blocker U73122 (10 μmol L^−1^) (n = 80 cells of 5 experiments). E, ${\mathrm{Ca}}_{i}^{2+}$ increase induced by bradykinin was abolished by the IP3R blocker araguspongin B (4 μmol L^−1^) (n = 80 cells of 5 experiments), but not by the RyR inhibitor ryanodine (10 μmol L^−1^) (n = 96 cells of 6 experiments). Traces are shown as mean ± SEM in corresponding experiments

Figure 1A illustrates that bradykinin induces a significant increase in ${\mathrm{Ca}}_{i}^{2+}$ in human cardiac c‐Kit^+^ progenitor cells. Changes in ${\mathrm{Ca}}_{i}^{2+}$ induced by bradykinin show a quick transient increase followed by a sustained low level increase in ${\mathrm{Ca}}_{i}^{2+}$ (Figure 1A), and the effect was fully prevented by the bradykinin type 2 receptor (B2R) antagonist HOE140 (30 nmol L^−1^), but not the B1R antagonist R715 (300 nmol L^−1^) (Figure 1B). Omitting extracellular Ca^2+^ (Ca^2+^‐free medium plus 1 mmol L^−1^ EGTA) abolished the sustained component without affecting the transient ${\mathrm{Ca}}_{i}^{2+}$ increase by bradykinin (Figure 1C). These results suggest that the sustained ${\mathrm{Ca}}_{i}^{2+}$ increase is dependent on Ca^2+^ influx and the transient ${\mathrm{Ca}}_{i}^{2+}$ increase results from Ca^2+^ release from intracellular calcium stores, similar to previous observations in cultured human foreskin fibroblasts^15^, ^16^, ^17^ but different from observations in afferent sensory neurons^18^ and vascular endothelial cells.^19^

To analyse the molecular mechanisms underlying Ca^2+^ signalling by bradykinin in human cardiac c‐Kit^+^ progenitor cells, we utilized pharmacological tools followed by siRNA approach to identify the molecules related to intracellular Ca^2+^ release from calcium stores and/or Ca^2+^ influx.

### 3.2 Effects of pharmacological inhibitors on Ca^2+^ signalling induced by bradykinin

Figure 1D,E shows that ${\mathrm{Ca}}_{i}^{2+}$ increase by bradykinin was abolished in cells pre‐treated with the PLC inhibitor U73122 (10 μmol L^−1^) or the inositol 1,4,5‐trisphosphate (IP3) receptor (IP3R) inhibitor araguspongin B^20^ (4 μmol L^−1^), but not ryanodine receptor (RyR) inhibitor ryanodine (10 μmol L^−1^). In addition, we found that cardiac RyR2 protein was expressed in human atria, but not in human cardiac c‐Kit^+^ progenitor cells (Supporting Information Figure S1). These results support the notion that in human cardiac c‐Kit^+^ progenitor cells, as in human skin fibroblasts,^16^ B2R activation stimulates PLC and generates IP3, which then activates ER IP3Rs to release Ca^2+^ followed by Ca^2+^ influx.

**Figure 2:**
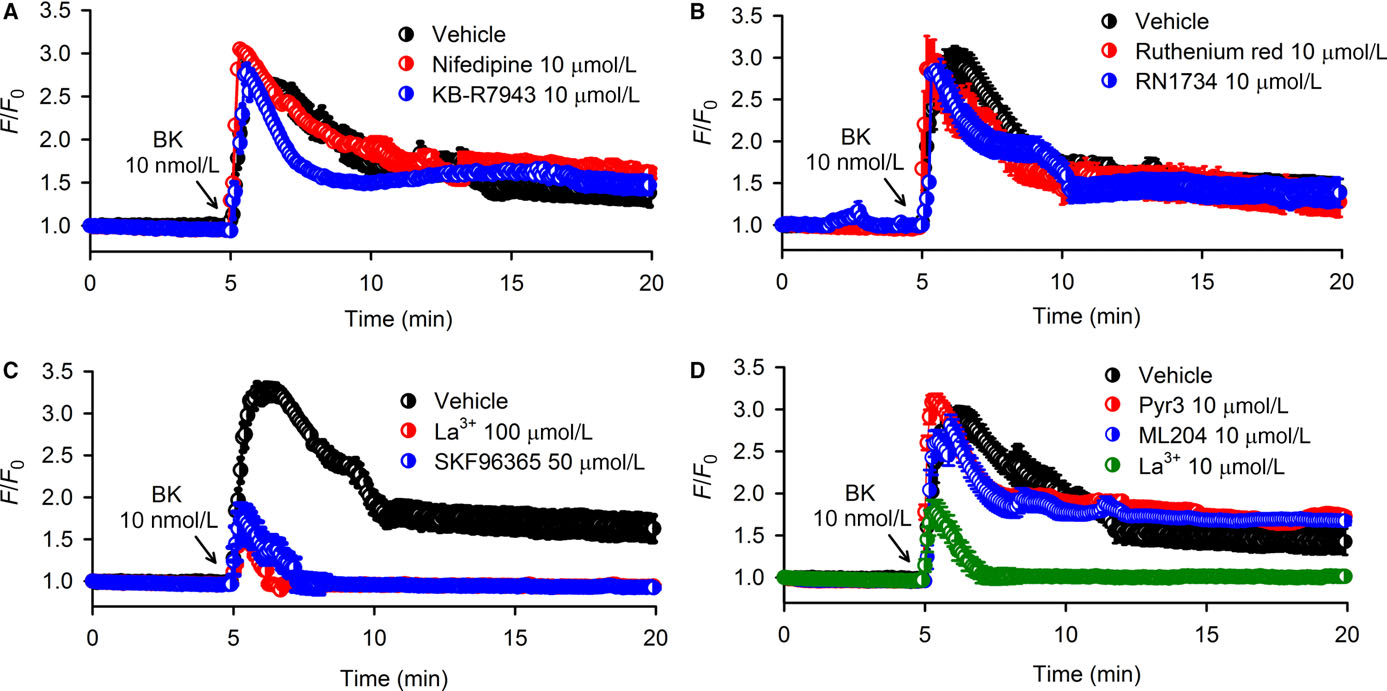
Effects of membrane channel blockers on ${\mathrm{Ca}}_{i}^{2+}$ increase by bradykinin (BK) in human cardiac c‐Kit^+^ progenitor cells. A, ${\mathrm{Ca}}_{i}^{2+}$ increase was not affected by the L‐type Ca^2+^ channel inhibitor nifedipine (10 μmol L^−1^) (n = 96 cells of 6 experiments) or NCX inhibitor KB‐R7943 (10 μmol L^−1^) (n = 96 cells of 6 experiments). B, ${\mathrm{Ca}}_{i}^{2+}$ increase was not affected by the TRPV2 channel blocker ruthenium red (10 μmol L^−1^) (n = 96 cells of 6 experiments) or the TRPV4 blocker RN1734 (10 μmol L^−1^) (n = 96 cells of 6 experiments). C, General TRP channel blocker La^3+^ (100 μmol L^−1^) (n = 96 cells of 6 experiments) or the non‐selective TRPC channel blocker SKF96365 (50 μmol L^−1^) (n = 80 cells of 5 experiments) inhibited the sustained Ca^2+^ influx, but not Ca^2+^ transient release induced by bradykinin. D, Selective SOCE channel blocker (La^3+^, 10 μmol L^−1^), but not selective TRPC3 blocker (Pyr3, 10 μmol L^−1^) (n = 96 cells of 6 experiments) or selective TRPC4 channel blocker (ML204, 10 μmol L^−1^) (n = 96 cells of 6 experiments), inhibited the sustained Ca^2+^ influx, but not Ca^2+^ transient release induced by bradykinin. Summarized traces are shown as mean ± SEM in corresponding experiments

To determine the possible ion channel involvement in Ca^2+^ influx by bradykinin, pharmacological blockers for different channels that mediate Ca^2+^ entry were tested in human cardiac c‐Kit^+^ progenitor cells. An earlier study reported that bradykinin‐induced Ca^2+^ influx was mediated by L‐type Ca^2+^ channels in cultured human skin fibroblasts.^15^ However, this is not the case in human cardiac c‐Kit^+^ progenitor cells, as bradykinin‐induced ${\mathrm{Ca}}_{i}^{2+}$ increase was not affected by pre‐treatment with the L‐type Ca^2+^ channel blocker nifedipine (10 μmol L^−1^) (Figure 2A). This is supported by Western blot analysis which showed that cardiac Cav1.2 protein was expressed in human atria, but not in human cardiac c‐Kit^+^ progenitor cells (Supporting Information Figure S1). In addition, we tested whether the reverse‐mode of Na^+^/Ca^2+^ exchanger mediates the agonist‐induced Ca^2+^ entry by pre‐treating the cells with 10 μmol L^−1^ KB‐R7943 (an inhibitor of the reverse Na^+^/Ca^2+^ exchanger). The bradykinin‐induced ${\mathrm{Ca}}_{i}^{2+}$ increase was also not affected in these cells (Figure 2A), suggesting that the reverse‐mode of Na^+^/Ca^2+^ exchanger is not involved in the ${\mathrm{Ca}}_{i}^{2+}$ increase.

Our previous study demonstrated that functional TRPV2 and TRPV4 channels were predominantly expressed in human cardiac c‐Kit^+^ progenitor cells.^11^ However, TRPV2 and TRPV4 channels are not involved in bradykinin‐induced ${\mathrm{Ca}}_{i}^{2+}$ increase because the ${\mathrm{Ca}}_{i}^{2+}$ increase was not affected by the TRPV2 blocker ruthenium red (10 μmol L^−1^) or the TRPV4 blocker RN1734 (10 μmol L^−1^) (Figure 2B). In cells pre‐treated with the broad spectrum TRP channel blocker La^3+^ (100 μmol L^−1^) or the non‐selective TRPC channel blocker SKF96365 (50 μmol L^−1^), bradykinin‐induced a transient ${\mathrm{Ca}}_{i}^{2+}$ increase (Figure 2C), suggesting that the Ca^2+^ influx is mediated by TRPC channels.

We then tested the selective TRPC3 blocker Pyr3^21^ and the selective TRPC4 blocker ML204;^22^ however, neither Pyr3 (10 μmol L^−1^) nor ML204 (10 μmol L^−1^) affected the Ca^2+^ activity induced by bradykinin (Figure 2D). This implies that TRPC3 and TRPC4 are not involved in mediating bradykinin‐induced Ca^2+^ influx. La^3+^ is a well‐known TRP channel blocker; however, a recent study reported that a low concentration of La^3+^ at 10 μmol L^−1^ can selectively block SOCE channel.^23^ We therefore used 10 μmol L^−1^ La^3+^ to test whether SOCE channel is involved in mediating the bradykinin‐induced Ca^2+^ influx. The results (Figure 2D) show that 10 μmol L^−1^ La^3+^ fully inhibited bradykinin‐induced Ca^2+^ influx without affecting ER IP3R Ca^2+^ release, indicating that bradykinin‐induced Ca^2+^ influx is mediated by SOCE channel in human cardiac c‐Kit^+^ progenitor cells.

### 3.3 Effects of siRNAs targeting IP3Rs and TRPC channels on Ca^2+^ signalling by bradykinin

To further determine the molecular identities of proteins involved in bradykinin‐mediated Ca^2+^ signalling in human cardiac c‐Kit^+^ progenitor cells, ${\mathrm{Ca}}_{i}^{2+}$ was determined in cells transfected with siRNA targeting B2R, IP3R1, IP3R2, IP3R3, TRPC1, TRPC3 or TRPC4, and also the SOCE channel components STIM1 (Stromal interaction molecule 1) and Orai1 (calcium release‐activated calcium channel protein 1). Genes and proteins of B2R, IP3R1, IP3R2, IP3R3, TRPC1, TRPC3, TRPC4, Orai1 and STIM1 were remarkably reduced in cells transfected with 10 and 40 nmol L^−1^ of the corresponding siRNAs (n = 5, *P* < .01 vs control siRNA) (Supporting Information Figures S2‐S4).

**Figure 3:**
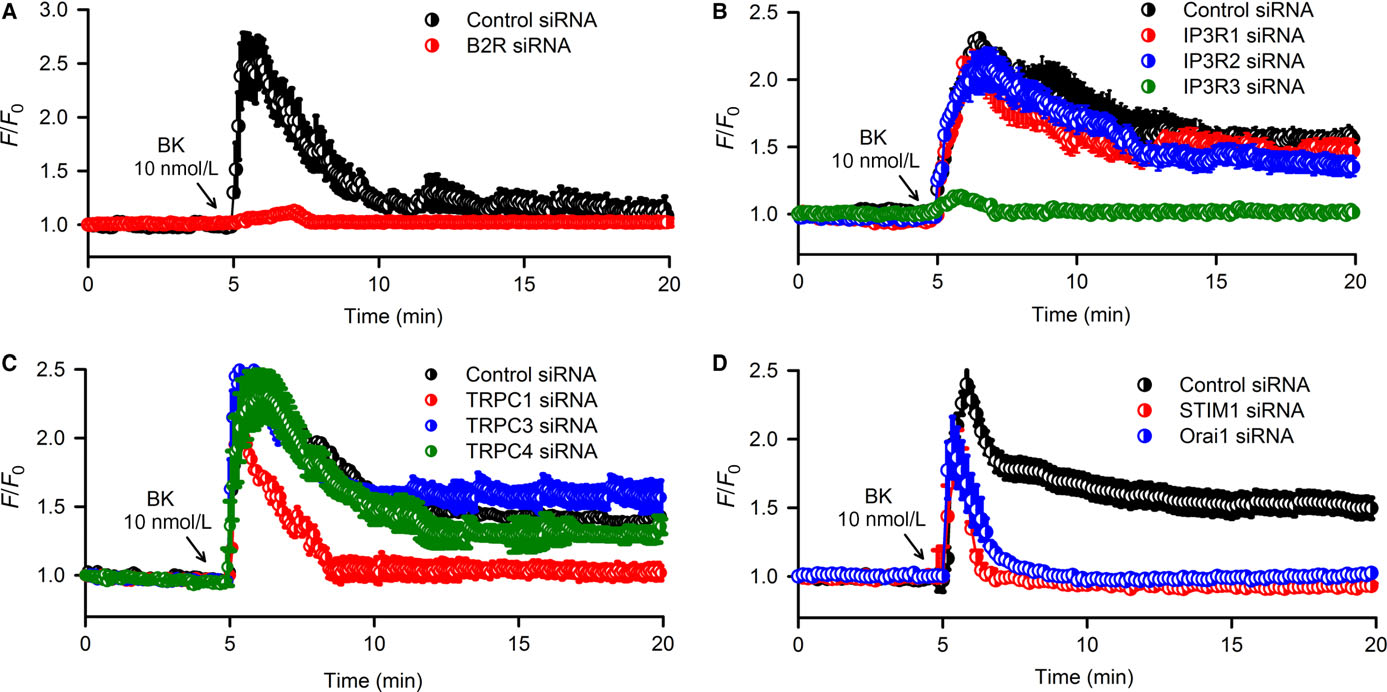
Effects of silencing related genes with corresponding siRNAs on ${\mathrm{Ca}}_{i}^{2+}$ increase induced by bradykinin (BK). A, Bradykinin did not induce ${\mathrm{Ca}}_{i}^{2+}$ increase in cells with silenced B2R (n = 80 cells of 5 experiments). B, Silencing IP3R3, but not IP3R1 or IP3R2, prevented the ${\mathrm{Ca}}_{i}^{2+}$ increase induced by bradykinin (n = 80 cells of 5 experiments). C, Silencing TRPC1, but TRPC3 or TRPC4, prevented the Ca^2+^ influx without affecting transient Ca^2+^ release induced by bradykinin (n = 96 cells of 6 experiments). D, Silencing Orai1 or STIM1 prevented the Ca^2+^ influx without affecting transient Ca^2+^ release induced by bradykinin (n = 96 cells of 6 experiments). Summarized traces are shown as mean ± SEM in corresponding experiments

Bradykinin could not induce ${\mathrm{Ca}}_{i}^{2+}$ increase in cells with silenced B2R (Figure 3A). Interestingly, bradykinin‐induced ${\mathrm{Ca}}_{i}^{2+}$ increase was significant in cells with silenced IP3R1 or IP3R2, but not in cells with silenced IP3R3 (Figure 3B). This indicates that the bradykinin‐induced ${\mathrm{Ca}}_{i}^{2+}$ increase in human cardiac c‐Kit^+^ progenitor cells is initiated by activating ER IP3R3 to release Ca^2+^ from intracellular calcium stores followed by Ca^2+^ influx.

Bradykinin‐induced Ca^2+^ influx through TRPC channel was further tested in cells with silenced TRPC1, TRPC3 or TRPC4 (Figure 3C), in which the Ca^2+^ influx was abolished only in cells transfected with TRPC1 siRNA, but not TRPC3 siRNA or TRPC4 siRNA.

Our recent study demonstrated that TRPC1, Orai1 and STIM1 are components of SOCE channel complex in human cardiac c‐Kit^+^ progenitor cells.^13^ If bradykinin‐induced Ca^2+^ influx is mediated solely by TRPC1 channel, the effect would not be affected in cells with silenced Orai1 or STIM1. However, in the cells with silenced Orai1 or STIM1, as in the cells with silenced TRPC1, bradykinin only induced a transient ER IP3R3 Ca^2+^ release without a subsequent Ca^2+^ influx. These results indicate that bradykinin‐induced Ca^2+^ influx is mediated by SOCE channel and depends on the integrity of channel complex.

### 3.4 Effects of silencing molecules of IP3Rs or SOCE on cell cycling progression and mobility

We have recently demonstrated that SOCE channel participates in regulating cell growth and migration,^13^ and bradykinin promotes cell cycling progression and mobility in cultured human cardiac c‐Kit^+^ progenitor cells.^10^ To investigate whether the stimulation of cell cycling progression and mobility by bradykinin is related to Ca^2+^ signalling. The effects of bradykinin on cell proliferation and migration were determined in cells with silenced IP3Rs and components molecules of SOCE channel.

**Figure 4:**
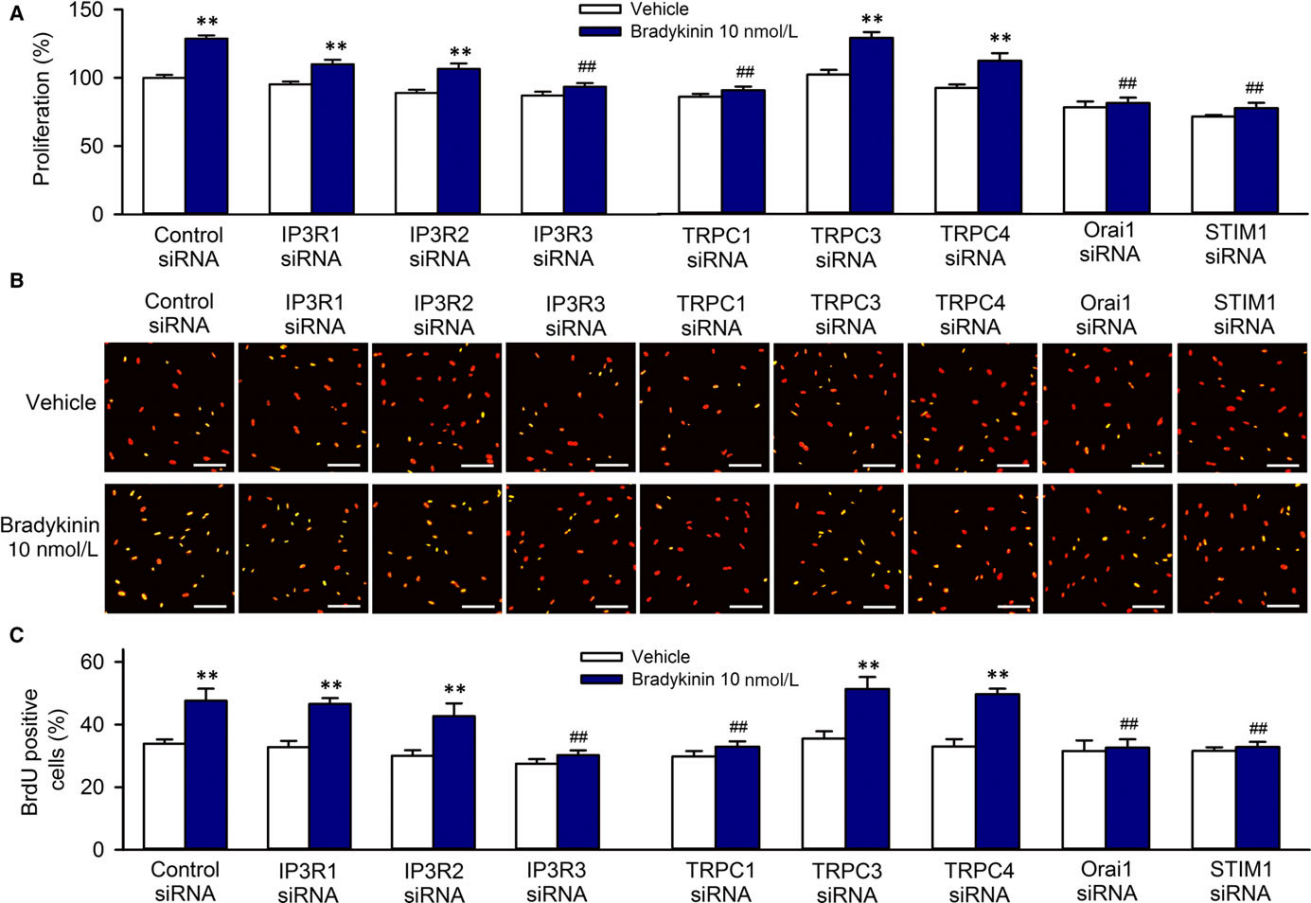
Effects of silencing IP3Rs or components of SOCE channel on cell growth and migration induced by bradykinin (BK). A, Cell proliferation determined with MTT assay in cells transfected with 40 nmol L^−1^ siRNAs targeting IP3R1, IP3R2, IP3R3, TRPC1, TRPC3, TRPC4, Orai1 or STIM1 in the absence or presence of 10 nmol L^−1^ bradykinin. B, Images of BrdU incorporation in cells transfected with 40 nmol L^−1^ siRNAs targeting IP3R1, IP3R2, IP3R3, TRPC1, TRPC3, TRPC4, Orai1 or STIM1 in the absence or presence of 10 nmol L^−1^ bradykinin. The proliferative cells show yellow colour from the merging of BrdU green staining with Propidium Iodide (PI) red nuclei staining. C, Percentage values of BrdU incorporation in cells transfected with 40 nmol L^−1^ siRNAs targeting IP3R1, IP3R2, IP3R3, TRPC1, TRPC3, TRPC4, Orai1 or STIM1 in the absence or presence of 10 nmol L^−1^ bradykinin. N = 5 experiments, **P < .01 vs control siRNA, ^##^ *P* < .01 vs control siRNA with 10 nmol L^−1^ bradykinin

Figure 4 illustrates the effects of bradykinin on cell proliferation determined by MTT (Figure 4A) and BrdU (Figure 4B,C) assays in cells transfected with corresponding siRNAs. Bradykinin 10 nmol L^−1^ significantly stimulated cell growth in cells with silenced IP3R1, IP3R2, TRPC3 or TRPC4 (n = 5 for each group, *P* < .01 vs vehicle) but had no such effect in cells with silenced IP3R3, TRPC1, Orai1 or STIM1 (n = 5 for each group, *P* = NS vs vehicle; *P* < .01 vs control siRNA with bradykinin 10 nmol L^−1^). These results indicate that the enhancement of cell growth by bradykinin in human cardiac c‐Kit^+^ progenitor cells is related to the Ca^2+^ signalling.

**Figure 5:**
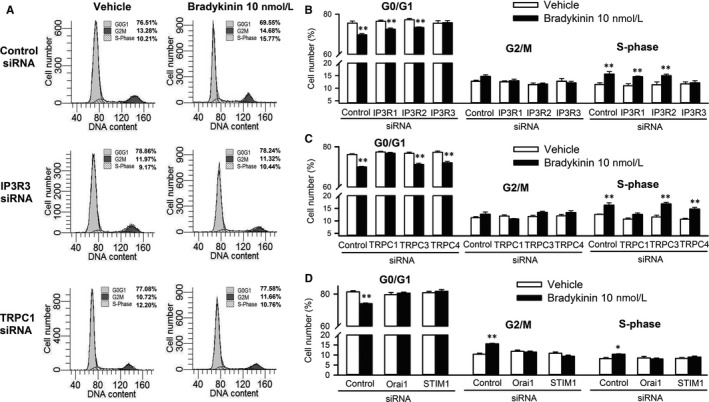
Effects of silencing IP3Rs or components of SOCE channel on cell cycling progression induced by bradykinin (BK). A, Representative flow cytometry graphs in cells transfected with control, IP3R3 or TRPC1 siRNA (40 nmol L^−1^) in the absence or presence of 10 nmol L^−1^ bradykinin. B, Percentage values of cell population at different cycling phases in cells transfected with control, IP3R1, IP3R2 or IP3R3 siRNA (40 nmol L^−1^) in the absence or presence of 10 nmol L^−1^ bradykinin. C, Percentage values of cell population at different cycle phases in cells transfected with control, TRPC1, TRPC3 or TRPC4 siRNA (40 nmol L^−1^) in the absence or presence of 10 nmol L^−1^ bradykinin. D, Percentage values of cell population at different cycle phases in cells transfected with control, Orai1 or STIM1 siRNA (40 nmol L^−1^) in the absence or presence of 10 nmol L^−1^ bradykinin treatment. N = 5, experiments *P < .05, **P < .01 vs control siRNA

The cell cycling progression was then determined by flow cytometry analysis in cells with silenced IP3Rs, TRPCs, STIM1 or Orai1 in the absence or presence of bradykinin (Figure 5). Bradykinin significantly promoted cell cycling progression by decreasing G0/G1 population and increasing S‐phase population in cells transfected with control, IP3R1, IP3R2, TRPC3 or TRPC4 siRNA (40 nmol L^−1^), but not in cells transfected with IP3R3, TRPC1, STIM1 or Orai1 siRNA. These results indicate that bradykinin‐induced promotion of cell cycling progression is related to ${\mathrm{Ca}}_{i}^{2+}$ increase mediated by IP3R3 and SOCE channel in human cardiac c‐Kit^+^ progenitor cells.

**Figure 6:**
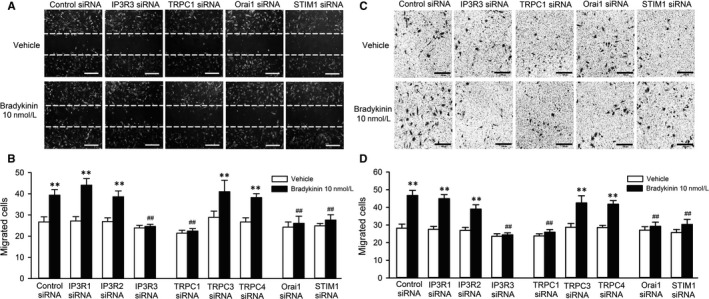
Effects of silencing IP3Rs or components of SOCE channel on migration induced by bradykinin (BK). A, Representative images of wound‐healing assay in cells transfected with control, IP3R3, TRPC1, Orai1 or STIM1 siRNA in the absence or presence of 10 nmol L^−1^ bradykinin treatment for 8 h. B, Mean values of cell number migrated into the acellular area in cells transfected corresponding siRNAs in the absence and presence of 10 nmol L^−1^ bradykinin. C, Images of migrated cells on the lower surface membrane in transwell assay in cells transfected with control, IP3R3, TRPC1, Orai1 or STIM1 siRNA in the absence or presence of 10 nmol L^−1^ bradykinin treatment for 8 h. D, Mean number of migrated cells on the lower surface membrane in cells transfected corresponding siRNAs in the absence or presence of 10 nmol L^−1^ bradykinin. N = 6 experiments, **P < .01 vs control siRNA, ^##^ *P* < .01 vs control siRNA with 10 nmol L^−1^ bradykinin

The effects of bradykinin on cell migration were determined with wound‐healing assay (Figure 6A,B) and transwell assay (Figure 6C,D) in cells transfected with 40 nmol L^−1^ control, IP3R1, IP3R2, IP3R3, TRPC1, TRPC3, TRPC4, Orai1 or STIM1 siRNA molecules. The cells migrated to the acellular area or the lower membrane surface of transwell were increased by bradykinin in cells transfected with control, IP3R1, IP3R2, TRPC3 or TRPC4 siRNA, but not in cells transfected with IP3R3, TRPC1, Orai1 or STIM1 siRNA. These results indicate that cell migration induced by bradykinin is related to ${\mathrm{Ca}}_{i}^{2+}$ increase via both IP3R3 and SOCE channel in human cardiac c‐Kit^+^ progenitor cells.

### 3.5 Ca^2+^ signalling by bradykinin and intracellular signal molecules is involved in regulating cell cycling progression and migration

**Figure 7:**
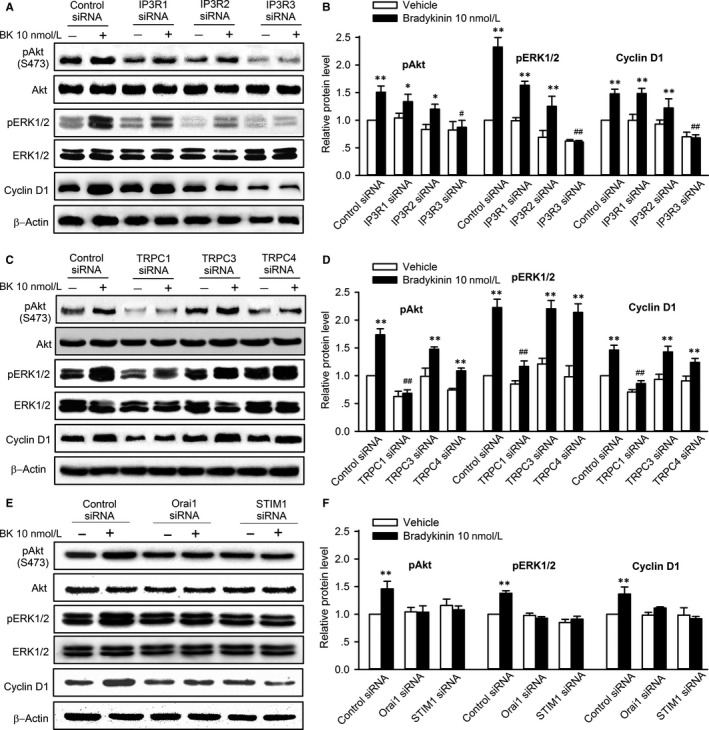
Effects of silencing IP3Rs or components of SOCE channel on increase of pAkt, pERK1/2 and cyclin D1 by bradykinin (BK). A, Western blots show that bradykinin (10 nmol L^−1^) increased pAkt, pERK1/2 or cyclin D1 expression in cells transfected with control, IP3R1 and IP3R2 siRNA, but not with IP3R3 siRNA. B, Relative protein levels of pAkt, pERK1/2 and cyclin D1 in cells transfected with corresponding IP3R siRNAs. C, Western blots show that bradykinin (10 nmol L^−1^) increased pAkt, pERK1/2 and cyclin D1 level in cells transfected with control, TRPC3 or TRPC4 siRNA, but not with TRPC1 siRNA. D, Relative protein levels of pAkt, pERK1/2 and cyclin D1 in cells transfected with control, TRPC1, TRPC3 or TRPC4 siRNA. E, Western blots show that bradykinin (10 nmol L^−1^) increased pAkt, pERK1/2 and cyclin D1 level in cells transfected with control siRNA, but not with Orai1 or STIM1 siRNA. F, Relative protein levels of pAkt, pERK1/2 and cyclin D1 in cells transfected with control, Orai1 or STIM1 siRNA. N = 5, **P < .01 vs control siRNA treated with vehicle, ^##^ *P* < .01 vs control siRNA with 10 nmol L^−1^ bradykinin

Our recent study has showed that activation of pAkt, pERK1/2 and cyclin D1 is involved in regulation of proliferation and/or migration by bradykinin in human cardiac c‐Kit^+^ progenitor cells.^10^ To determine whether pAkt, pERK1/2 and cyclin D1 expression is mediated by ${\mathrm{Ca}}_{i}^{2+}$ increase, these signal molecules were determined in cells with silenced IP3Rs, TRPCs and SOCE components. Figure 7 illustrates the Western blot analysis of pAkt, pERK1/2 and cyclin D1 in cells transfected with IP3Rs, TRPCs or SOCE siRNAs. The increased expression of pAkt, pERK1/2 and cyclin D1 by bradykinin was seen in cells transfected with control, IP3R1, IP3R2, TRPC3 or TRPC4 siRNA, but not in cells transfected with IP3R3, TRPC1, Orai1 or STIM1 siRNA. These results support the notion that stimulation of cell cycling progression and migration by bradykinin is related to activating pAkt, pERK1/2 and cyclin D1 by increasing ${\mathrm{Ca}}_{i}^{2+}$ increase in human cardiac c‐Kit^+^ progenitor cells.

## 4 DISCUSSION

Although human cardiac c‐Kit^+^ progenitor cells is a promising cell source for repairing ischaemic cardiomyopathy,^4^, ^5^ one of limitations for this regenerative therapy is the poor survival rate of transplanted cells in the host myocardium.^6^, ^7^, ^8^ Previous studies have explored different strategies to enhance the survival rate of the donor cells using growth factor genes^24^ or preconditioning donor stem cells with different interventions including nitric oxide donor,^25^ HO‐1 inducer CoPP^26^ and exposure to hypoxia.^27^, ^28^ Our recent report^10^ and the present study demonstrated that the endogenous peptide bradykinin can initiate proliferation and migration of human cardiac c‐Kit^+^ progenitor cells.

The present study has demonstrated that stimulation of cell cycling progression and migration by bradykinin is related to ${\mathrm{Ca}}_{i}^{2+}$ increase via Ca^2+^ release‐induced Ca^2+^ influx mechanism: (1) bradykinin binds to B2R to stimulate PLC and generate IP3, (2) IP3 activates ER IP3R3 to release Ca^2+^ and (3) Ca^2+^ release induces a reduced ER Ca^2+^ level and activates SOCE channel to mediate Ca^2+^ influx. The bradykinin‐induced Ca^2+^ signalling plays a crucial role in regulating cell cycling progression and mobility via activating pAkt, pERK1/2 and cyclin D1 in human cardiac c‐Kit^+^ progenitor cells (Supporting Information Figure S5); however, we cannot rule out that other signalling pathways, such as NAADP and TPC1‐2, could be involved in bradykinin‐induced intracellular Ca^2+^ release.

It is well recognized that cytosolic free Ca^2+^ is a secondary messenger which plays crucial roles in regulating many cellular activities, for example, muscle contraction, gland secretion, cell growth, differentiation, migration, survival and apoptosis.^29^, ^30^, ^31^ In endothelial progenitor cells, Ca^2+^ oscillations play a key role in stimulating colony cells proliferation and tubulogenesis.^32^ In a number of cardiac progenitor and stem cells, Ca^2+^ signalling mediates cardiovascular regeneration.^9^, ^33^ Bradykinin is an endogenous peptide that not only participates in the mitogenesis process,^34^ myocardial^35^, ^36^, ^37^ and neuronal^38^, ^39^ protection, but also mediates respiratory allergic reactions, septic shock, acquired angioedema etc.^40^ These physiological effects or pathophysiological responses may result from different Ca^2+^ signalling pathways in different types of cells/organs. In bovine coronary artery, bradykinin‐induced vasodilation is related to endothelial ${\mathrm{Ca}}_{i}^{2+}$ increase mediated by cyclic ADP ribose‐RyRs Ca^2+^ signalling pathway, but not PLC‐IP3Rs Ca^2+^ signalling pathway, because the PLC inhibitor U73122 and the IP3R inhibitor 2‐aminoethoxydiphenyl borate do not affect the ${\mathrm{Ca}}_{i}^{2+}$ increase induced by bradykinin.^41^ However, an earlier study demonstrated that bradykinin induces an increase in inositol 1,4,5‐triphosphate (IP3) in neonatal rat cardiomyocytes^42^ and bradykinin‐mediated short nocifensive responses is related to activating PLC, followed by Orai1 in afferent sensory neurons.^18^

In rat C6 glioma cells, bradykinin triggers Ca^2+^ influx, resulting in calcium store Ca^2+^ release, which is associated with nitric oxide generation for regulating permeability of blood‐tumour barrier,^43^ while in primary cultured rat brain microvascular endothelial cells, bradykinin‐triggered Ca^2+^ influx‐induced Ca^2+^ release is involved in regulation of the junction protein claudin‐5.^19^ A recent study shows that Ca^2+^ signalling induced by bradykinin results from initial Ca^2+^ release from ER IP3Rs followed by Ca^2+^ entry through Ca^2+^ release‐activated channels in normal pancreatic stellate cells.^44^ This is similar to the earlier report in human foreskin fibroblasts,^16^ and the present observation in human cardiac c‐Kit^+^ progenitor cells.

Nonetheless, most of the previous reports on various types of cells were obtained using pharmacological tools, and the molecular identities of Ca^2+^ signal pathway involved in bradykinin‐mediated ${\mathrm{Ca}}_{i}^{2+}$ are not fully understood. Although in this study, we found some molecules involved in bradykinin‐induced Ca^2+^ signalling and modulation of cell cycling progression and migration using both pharmacological tools and biochemical/molecular biological approaches, we cannot completely exclude the involvement of other potential molecules that would mediate Ca^2+^ signalling by bradykinin in c‐Kit^+^ progenitor cells.

Our previous study has shown that B2Rs, but not B1Rs, are expressed in human cardiac c‐Kit^+^ progenitor cells.^10^ Mediation of Ca^2+^ signalling by B2Rs is demonstrated in cells pre‐treated with HOE140 or transfected with B2R siRNA, in which bradykinin no longer induces ${\mathrm{Ca}}_{i}^{2+}$ increase. Involvement of PLC and IP3Rs in Ca^2+^ signalling is confirmed by the PLC inhibitor U73122 and the IP3R blocker raguspongin B. Ryanodine did not affect the Ca^2+^ signals, supporting the previous report that RyRs are not expressed in human cardiac c‐Kit^+^ progenitor cells.^9^

Inositol 1,4,5‐trisphosphate receptors (IP3Rs) play a key role in intracellular calcium signalling.^45^ It is not only the primary cytosolic target responsible for the initiation of intracellular calcium (Ca^2+^) signalling, but can also control apoptosis, intracellular pH, the initiation and regulation of neuronal Ca^2+^ signalling, exocytosis and gene expression by coupling with modulatory protein.^46^ There are three types of IP3R subunits (IP3R1, IP3R2 and IP3R3) expressed in mammalian cells, which co‐assemble to form homo‐ or heterotetrameric IP3R channels^47^ and mediate Ca^2+^ release from ER/SR in different types of cells. Although the three types of IP3Rs are expressed in human cardiac c‐Kit^+^ progenitor cells, we found that only IP3R3 mediates the bradykinin‐induced Ca^2+^ release and the subsequent Ca^2+^ influx through SOCE channel.

SOCE channel is a complex composed of STIM1, Orai1 and also TRPC1. STIM1 functions as an ER Ca^2+^ sensor, and Orai1 is a pore subunit located on the plasma membrane.^48^ Although there exist arguments on whether TRPC1 is a component of SOCE channel,^49^ our recent results from human cardiac c‐Kit^+^ progenitor cells^13^ support the notion that TRPC1 works together with Orai1 to form discrete STIM1‐gated SOCE channel, mediate distinct Ca^2+^ signals and regulate specific cellular functions.^50^, ^51^, ^52^

The present study shows that bradykinin‐induced Ca^2+^ influx in c‐Kit^+^ progenitor cells results from PLC‐IP3R3 ER‐Ca^2+^ release and activates the STIM1‐Orai1‐TRPC1 complex of SOCE channel to mediate extracellular Ca^2+^ entry. The involvement of TRPC1 in SOCE channel is demonstrated by the fact that inhibition of TRPC1 with the silencing TRPC1 abolishes the bradykinin‐induced Ca^2+^ influx. Similar results were observed in cells with silenced STIM1 or Orai1, in which bradykinin only induces a Ca^2+^ transient release without subsequent Ca^2+^ influx. This indicates that bradykinin‐induced Ca^2+^ influx relies on the SOCE channel as a STIM1‐Orai1‐TRPC1 complex in human cardiac c‐Kit^+^ progenitor cells.

In Ca^2+^ signalling, very low cytoplasmic Ca^2+^ concentration can increase in a specific manner to trigger downstream cellular events, and every cell type in the human body utilizes some form of Ca^2+^ signalling to function or survive.^29^ Ferreira‐Martins and colleagues reported that spontaneous Ca^2+^ oscillations promote human cardiac c‐Kit^+^ progenitor cell proliferation.^9^ We have previously found that Ca^2+^ signalling‐related channels (SOCE, TRPV2 and TRPV4) are involved in cell cycling progression and migration in human cardiac c‐Kit^+^ progenitor cells.^11^, ^13^ It has been demonstrated that division of c‐Kit^+^ cardiac stem cells in the mouse is promoted by spontaneous Ca^2+^ spikes, which dictate the pattern of stem cell replication and the generation of myocyte progeny at all phases of prenatal life and up to 1 day after birth.^53^

The endogenous peptide bradykinin has an extensive range of biological activities in different organs, including regulation of mitogenesis in different types of cells.^54^ Our previous study showed that bradykinin stimulated cell cycling progression and migration via activating pAkt and pERK1/2 as well as expression of cyclin D1 in human cardiac c‐Kit^+^ progenitor cells.^10^ The present study demonstrates the novel molecular mechanisms of bradykinin that involves Ca^2+^ signalling mediated by PLC‐IP3R3 ER‐Ca^2+^ release followed by Ca^2+^ influx through SOCE channel (STIM1‐Orai1‐TRPC1 complex); the cell growth, migration as well as pAkt, pERK1/2 and cyclin D1 are no longer increased by bradykinin in cells with silenced IP3R3, STIM1, Orai1 or TRPC1.

In the present study, we did not explore why IP3R3s, but not IP3R1s and IP3R2s, are activated by bradykinin. However, this limitation would not affect the conclusion that bradykinin is coupled with B2R to activate PLC and generate IP3, which then stimulates ER IP3R3 to release Ca^2+^ followed by Ca^2+^ influx through SOCE channel in human cardiac c‐Kit^+^ progenitor cells.

Collectively, cell proliferation and migration of cardiac c‐Kit^+^ progenitor cells are an essential cellular function for the heart development^53^ and cardiac repair, in which cardiac stem/progenitor cells are required to migrate to the infarcted myocardial zone and proliferate.^55^, ^56^ The present study demonstrates that bradykinin‐induced Ca^2+^ signalling via stimulating ER IP3R3 to release Ca^2+^ followed by Ca^2+^ influx through SOCE channel initiates Akt and ERK1/2 phosphorylation and cyclin D1 expression and promotes cell proliferation and migration, suggesting that bradykinin may be useful for preconditioning cardiac progenitor cells to promote myocardial repair.

## CONFLICT OF INTEREST

The authors confirm that there are no conflict of interests.
