# Supplementary material for: Bradykinin‐mediated Ca2+ signalling regulates cell growth and mobility in human cardiac c‐Kit+ progenitor cells
Source: J Cell Mol Med. 2018 Aug 17;22(10):4688–99. doi: 10.1111/jcmm.13706 (PMC6156395; doi:10.1111/jcmm.13706)
Supplement: Supplementary file 1 [file JCMM-22-4688-s001.pdf]

DOI: 10.1111/jcmm.13706 wileyonlinelibrary.com/journal/jcmm

Supporting Information

Supplementary Materials and Methods

Experimental solutions and reagents

Tyrode's solution contains (in mM) 140 NaCl, 5 KCl, 1.0 $MgCl_2$, 1.8 $CaCl_2$, 10 HEPES, and 10 glucose with pH adjusted to 7.3 using NaOH. Fetal bovine serum (FBS), α-MEM (Minimum Essential Medium Eagle-Alpha modification with Nucleoside), penicillin and streptomycin were purchased from Hyclone (Logan, Utah, USA). Fluo-4 AM, Propidium iodide (PI) and Lipofectamine 2000 were purchased from Thermo Fisher Scientific Inc. (Waltham, MA, USA). Bradykinin, HOE140, 5-bromo-2-deoxyuridine (BrdU), La^{3+}, and monoclonal anti-BrdU antibody were from Sigma-Aldrich (St. Louis, MO, USA). Anti-IP3R1 (07-1210), IP3R2 (AB3000) and IP3R3 (AB9076) antibodies, polyvinylidene difluoride (PVDF) transfer membrane were from Millipore (Billerica, MA, USA). Cell culture dishes or plates and transwell polycarbonate membrane cell culture inserts were obtained from Corning Inc. (Corning, NY, USA). Anti-TRPC1 (sc-133076), TRPC3 (sc-514670), Orai1 (sc-68895), cyclin D1 (sc-20044), cyclin E (sc-377100) and β-actin antibodies, araguspongin B and siRNA molecules products were from Santa Cruz Biotechnology (Dallas, TX, USA). TRPC4 (Ab131114) and Stim1 (Ab108994) were products of Abcam (Cambridge, UK). Akt (#4685S), phospho-Akt (Ser473) (#4060), p44/42 MAPK (ERK1/2) (#4695S), and phospho-p44/42 MAPK (ERK1/2) (Thr202/Tyr204) (#4370) were purchased from Cell Signaling Technology (Boston, Massachusetts, USA). SKF96365, U73122, RN-1734, ruthenium red, Pyr3, ML204, and GsMTx4 were obtained from Tocris Bioscience (Bristol, UK). Basic fibroblast growth factor (bFGF) and epidermal growth factor (EGF) were from Ebioscience (San Diego, CA, USA).

Cell proliferation assay

Cell proliferation was detected with 3-(4,5-dimethyl-2-thiazolyl)-2,5-diphenyltetrazolium bromide (MTT) and 5-bromo-2-deoxy uridine (BrdU) in human cardiac c-Kit^+ progenitor cells transfected with siRNAs targeting IP3Rs, TRPCs, SOCEs and IP3Rs for 60 h. For MTT assay, cells were seeded in 96-well plates at 3×10^3 cells per well and then were transfected with corresponding siRNAs with 40 nM for 60 h, then treated with 10 nM bradykinin or vehicle for additional 24 h. MTT stock solution 10 μl (5 mg/ml) was then added to each well, and the plates were incubated at 37 ^oC for additional 4 h. Dimethyl sulfoxide (150 μl) was added to dissolve the formazan crystals after removed the medium. The values of the optical density of the samples were read using a microplate spectrophotometer (Bio-Tek Instruments, Winooski, VT, USA).

Approximately 5×10^4 cells per well were plated on 6-well culture plates with a cover slip in each well for BrdU assay as described previously [10]. After transfected with corresponding siRNAs for 60 h, cells were incubated with 20 μM BrdU for 24h in the presence or absence of bradykinin 10 nM in the assay medium. After BrdU labeling, cells were fixed and immune stained using mouse anti-BrdU antibody (at 1:200, Sigma-Aldrich) and Alexa 488-conjugated anti-mouse secondary antibody (at 1:500, Invitrogen). Cell nucleus was also stained with Propidium iodide (PI). Images were captured using Leica SP5II laser scanning confocal microscopy (Leica, Germany), and the BrdU-positive cells were counted manually.

Cell mobility assay

The effects of bradykinin on human cardiac c-Kit$^+$ cells transfected with corresponding siRNA was determined with wound healing and transwell assay as described previously[11-14]. For wound healing assay, after the cells transfected with correspondence siRNA for about 72 h, a standard wound was created by scratching the cell monolayer with a 200 μl pipette and line makers were made to indicate the wound edges. The cell fragments were flushed with PBS for three times. Then the cells were treated with bradykinin or vehicle with FBS-free medium at 37°C for 8 h. The areas of the wound gap were photographed with a microscope (Olympus, Tokyo, Japan). The amount of migrated cells in the images was counted to assess cell migration ability under different conditions.

Transwell assay was applied with a modified Boyden chamber with 8 μm-pore polycarbonate membrane to determine cell migration to exclude potential contamination by cell proliferation. Briefly, the chambers were pre-coated with serum-free medium for at least 30 min. Then the medium was removed and approximately 1×10^4 cells transfected with corresponding siRNAs were plated into the upper chamber in 200 μl 1% FBS medium in the absence or presence of bradykinin 10 nM, and the lower chambers were added 600 μl 1% FBS medium. The plates were incubated at 37℃ in 5% CO_2 for 8 h. Then the chambers were washed with PBS, fixed with 4% paraformaldehyde for 20 min at room temperature, and stained with crystal violet for 15 min. After washing thoroughly with PBS to remove the crystal violet, non-migrated cells on the upper membranes were carefully wiped off with cotton swabs. The migrated cells from the lower surface of the membrane were photographed by microscopy and counted to evaluate the migration ability of human cardiac c-Kit$^+$ progenitor cells transfected with corresponding siRNAs and treated bradykinin.

Table S1. Human gene-specific primers for RT-PCR

DOI: 10.1111/jcmm.13706 wileyonlinelibrary.com/journal/jcmm

**Gene (Accession No.)**	**Primer sequences (5'-3')**	**Fragment Size, bp**
BDKRB2 (NM_ 000623)		
Forward	CCTCACTCACATCCCACTC	234
Reverse	CACGAACAGCACCCAGA	
ITPR1 (NM_002222)		
Forward	TGACGAGAACCTGCCCTAT	430
Reverse	TCCTTTCGCCATCTTGCT	
ITPR2 (NM_002223)		
Forward	GCAATCGTGTCTGTTCCA	332
Reverse	TCTTCAAGTCTCAGCATCG	
ITPR3 (NM_002224)		
Forward	GCCTACTATGAGAACCACACG	389
Reverse	CAGAAGAGCAATGAGATGAGAG	
TRPC1 (NM_003304)		
Forward	CTGGTATGAAGGGTTGGAAGA	451
Reverse	AAAGCAGGTGCCAATGAAC	
TRPC3 (NM_003305)		
Forward	ATGACAGTGATGCGGGAGA	430
Reverse	CCTCGTCGTAAGCGTAGAAGT	
TRPC4 (NM_016179)		
Forward	TGGATGATATTACCGTGGGT	345
Reverse	CTTCAAAATGTCCAGGAGCA	
Orai1 (NM_ 032790)		
Forward	GAGTTACTCCGAGGTGATGA	307
Reverse	GACCGAGTTGAGATTGTGC	
Stim1 (NM_003156)		
Forward	GCAGAGTTTTGCCGAATTG	499
Reverse	TGAGGTGATTATGGCGAGTC	
GAPDH (J02642)		
Forward	AACAGCGACACCCACTCCTC	258
Reverse	GGAGGGGAGATTCAGTGTGGT	

DOI: 10.1111/jcmm.13706 wileyonlinelibrary.com/journal/jcmm

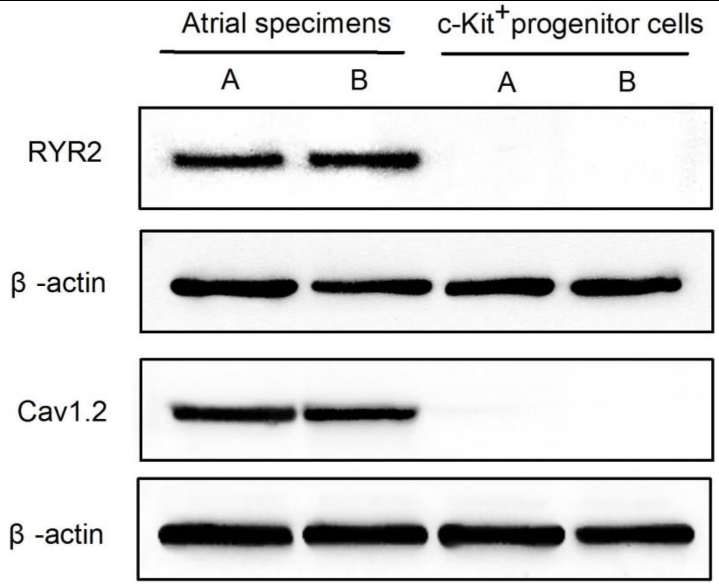

Figure S1. RYR2 and Cav1.2 expression in human atrial specimens and c-Kit^{+} progenitor cells. A and B represent two different specimens or two different c-Kit^{+} progenitor cells.

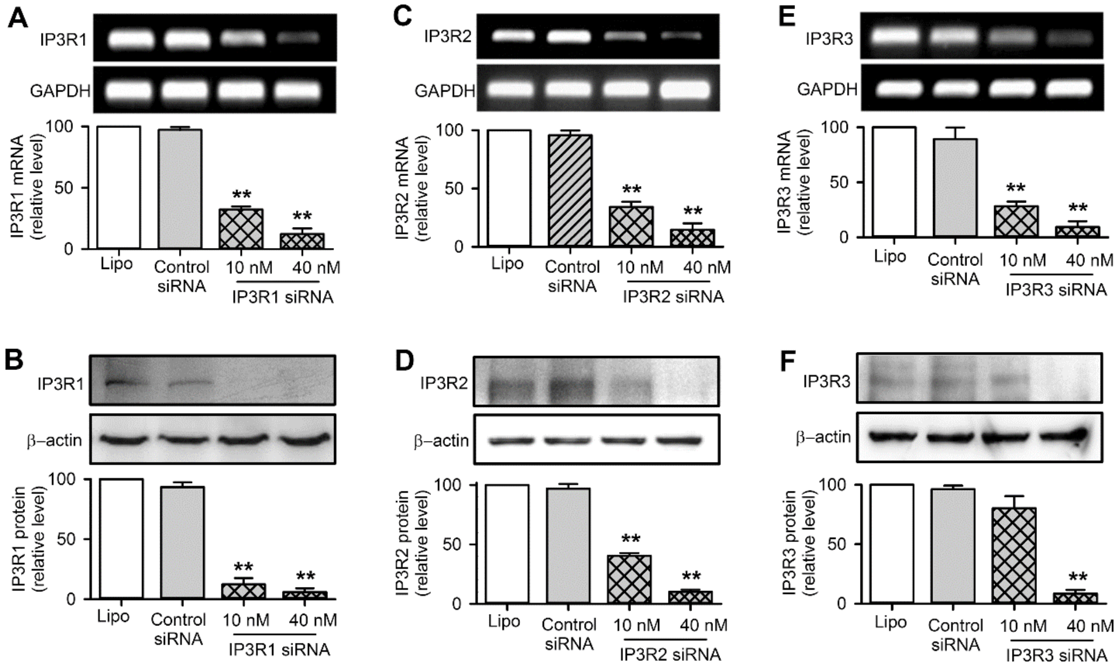

Figure S2. Silencing IP3R1, IP3R2 and IP3R3 in human cardiac c-Kit$^+$ progenitor cells transfected with corresponding siRNA. A: RT-PCR images and relative gene levels of IP3R1 in cells treated with Lipofectamine 2000 (Lipo), control siRNAs, or IP3R1 siRNA (10 and 40 nM) for 48 h. **B**: Western blots and relative protein levels of IP3R1 in cells treated with IP3R1 siRNA for 72 h. **C**: RT-PCR images and relative gene levels of IP3R2 in cells treated with IP3R2 siRNA for 48 h. **D**: Western blots and relative protein levels of IP3R2 in cells treated with IP3R2 siRNA for 72 h. **E**: RT-PCR images and relative gene levels of IP3R3 in cells treated with IP3R3 siRNA for 48 h. **F**: Western blots and relative protein levels of IP3R3 in cells treated with IP3R3 siRNA for 72 h. **P<0.01 vs. control siRNA.

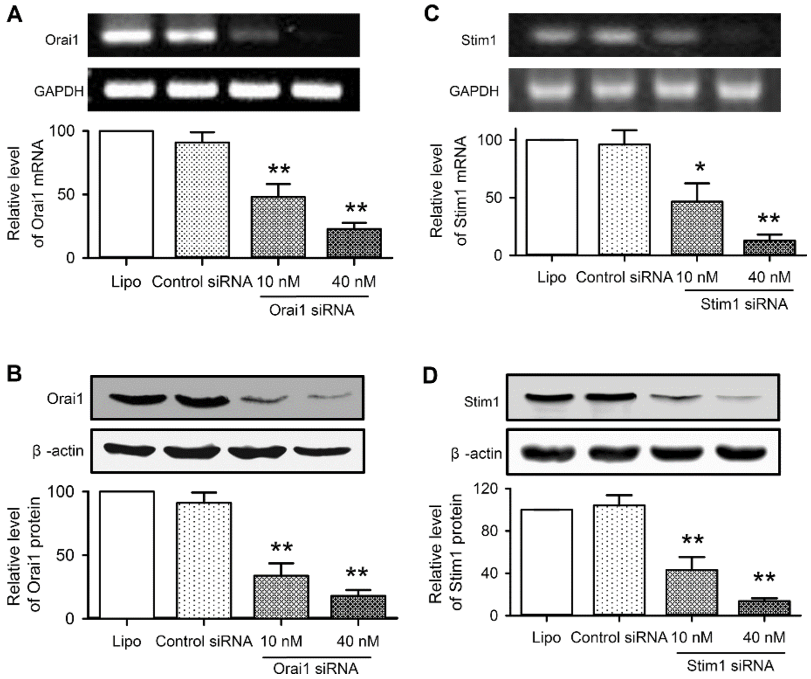

Figure S3. Silencing Orai1 and Stim1 in human cardiac c-Kit$^+$ progenitor cells transfected with corresponding siRNA. A: RT-PCR images and relative gene levels of Orai1 in cells treated with Lipofectamine 2000 (Lipo), control siRNAs, or Orai1 siRNA (10 and 40 nM) for 48 h. **B**: Western blots and relative protein levels of Orai1 in cells treated with Orai1 siRNA for 72 h. **C**: RT-PCR images and relative gene levels of Stim1 in cells treated with Stim1 siRNA for 48 h. **D**: Western blots and relative protein levels of Stim1 in cells treated with Stim1 siRNA for 72 h. $^{**}P < 0.01$ vs. control siRNA.

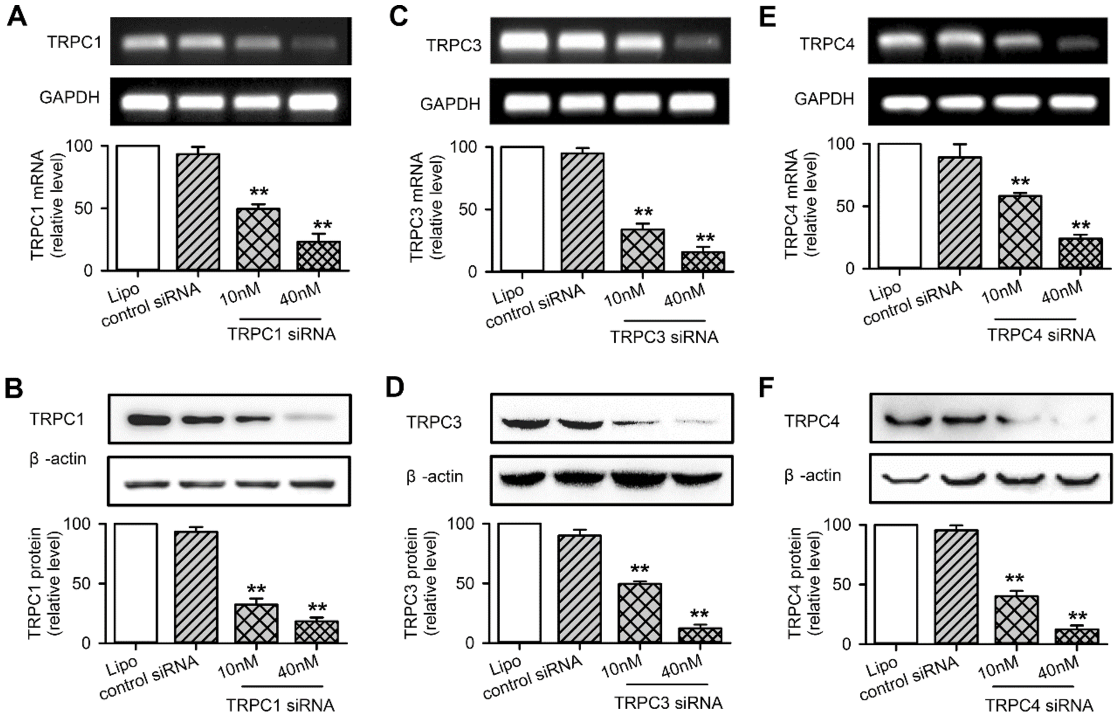

Figure S4. Silencing TRPC1, TRPC3 and TRPC4 in human cardiac c-Kit$^+$ progenitor cells transfected with corresponding siRNA. A: RT-PCR images and relative gene levels of TRPC1 in cells treated with Lipofectamine 2000 (Lipo), control siRNAs, or TRPC1 siRNA (10 and 40 nM) for 48 h. **B**: Western blots and relative protein levels of TRPC1 in cells treated with TRPC1 siRNA for 72 h. **C**: RT-PCR images and relative gene levels of TRPC3 in cells treated with TRPC3 siRNA for 48 h. **D**: Western blots and relative protein levels of TRPC3 in cells treated with TRPC3 siRNA for 72 h. **E**: RT-PCR images and relative gene levels of TRPC4 in cells treated with TRPC4 siRNA for 48 h. **F**: Western blots and relative protein levels of TRPC4 in cells treated with TRPC4 siRNA for 72 h. **$P < 0.01$ vs. control siRNA.

DOI: 10.1111/jcmm.13706 wileyonlinelibrary.com/journal/jcmm

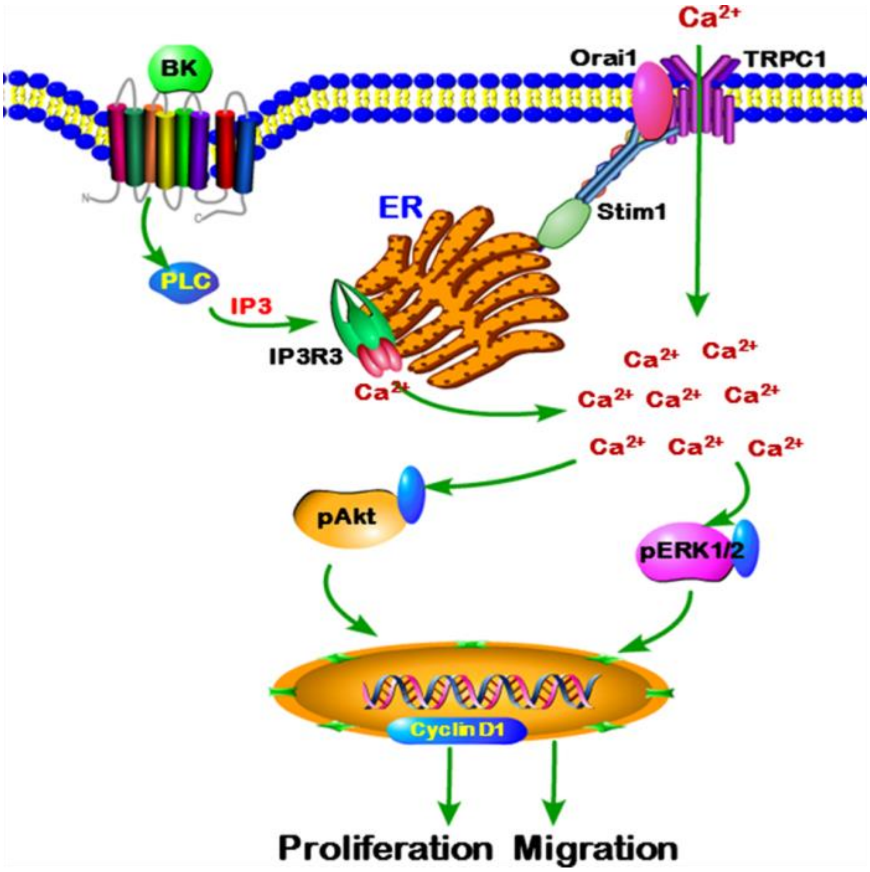

Figure S5. Schematic graph showing the molecular pathway of how bradykinin (BK) activates PLC, produces IP3, stimulates ER-IP3R1 Ca^{2+} release, and subsequently induces Ca^{2+} influx through SOCE channel. The $Ca^{2+}{}_i$ increase promotes cell growth and migration by increasing pAkt, pERK1/2, and cyclin D1 in human cardiac c-Kit$^+$ progenitor cells.
